# Comparative spatial cognition in wild Tanganyikan cichlids: navigation performance varies with home range and shelter availability

**DOI:** 10.1007/s10071-026-02064-2

**Published:** 2026-04-13

**Authors:** Zoë Goverts, Maëlan Tomasek, Alex Jordan

**Affiliations:** 1 Behavioural Evolution Research Group, Max Planck Institute of Animal Behavior, Konstanz, Germany; 2 LAboratoire de Psychologie Sociale et Cognitive, UMR6024, CNRS, UCA, Clermont-Ferrand, 63000 France

**Keywords:** Spatial cognition, Homing behaviour, Navigation, Landmark use, Path integration, Range size hypothesis, Behavioural ecology, Lake Tanganyika cichlids, Habitat structure, Comparative cognition

## Abstract

Spatial cognition varies widely even among closely related species, and identifying the ecological drivers of this variation remains challenging because species typically differ in multiple aspects of their ecology simultaneously. Here we performed wild experiments to compare spatial navigation in seven related species of shell-dwelling cichlids from Lake Tanganyika that share fundamental aspects of their ecology but vary in home range size and shelter availability. We displaced territorial males at successively greater distances (30 cm to 15 m) from shelters, using video tracking and automated 3D terrain reconstruction to measure return paths and homing success. We complemented this with onshore experiments examining landmark use and memory for shell locations. Of the seven species tested, three (*Lamprologus ornatipinnis*,* L. ocellatus*,* Neolamprologus meeli*) reliably returned to their home shells while four species (*N. multifasciatus*,* N. brevis*,* N. pulcher*,* Telmatochromis temporalis*) sought shelter in the nearest refuge. Among the navigating species, homing success declined with displacement distance, and long-distance performance (at 15 m) differed: *N. meeli* (largest range, fewest shelters) succeeded in 80% of trials, while *L. ocellatus* and *L. ornatipinnis* (smaller ranges, marginally more shelters) succeeded in only ~ 10% of trials. Arena experiments revealed that the two better-performing species in the field preferentially searched in areas previously associated with their home shell, with *N. meeli* showing additional use of experimental landmarks. Our findings provide a detailed comparative foundation for future work, highlighting how ecological context shapes spatial cognition in natural populations.

**Supplementary Information:**

The online version contains supplementary material available at 10.1007/s10071-026-02064-2.

## Introduction

Spatial cognition, which is the ability to encode, store, and use information about locations, routes, and landmarks, varies widely, even among closely related species (Fagan et al. 2013). Understanding the factors that drive this variation is challenging because species typically differ in multiple aspects of their ecology simultaneously. The range size hypothesis for example, posits that species with larger home ranges should have enhanced spatial abilities compared to those with smaller ranges (Spencer 2012). Yet under natural conditions range size rarely varies independently of other ecological factors: species with larger ranges often occupy different habitats, face different predation pressures, exploit different resources, and employ different social systems, and each of these factors could independently shape cognition (Healy and Rowe 2007; González-Forero and Gardner 2018).

Comparative studies may be most valuable when they document how multiple factors covary with cognitive performance, in turn providing a principled foundation for future experimental manipulations and mechanistic studies. At the same time, species that vary too greatly in their ecology can obfuscate relationships. For example, in a test of predictions from the range size hypothesis, the spatial memory of giant pandas (*Ailuropoda melanoleuca)*, a promiscuous species in which males occupy larger ranges than females, was compared to that of Asian small-clawed otters (*Aonyx cinereus*), a monogamous species with shared home ranges. Males outperformed females in pandas but not in otters, with differences in home range (i.e. the range size hypothesis) suggested as an explanation for the observed differences in spatial cognition (Perdue et al. 2011). While compelling, the multiple other differences among these species make it difficult to isolate the role of range size versus other factors affecting spatial memory. As such, comparative studies within closely related species groups that share most aspects of their ecology but differ in specific, quantifiable ways offer a powerful approach to identify ecological correlates of cognitive variation.

In this light, the explosive adaptive radiation of Lake Tanganyika cichlid fish offers a useful comparative system to explore variation in cognition without many of the confounds of other comparative systems (e.g. Tomasek et al. 2024). These small fish live in relatively constrained environments defined by the presence of fossilized snail shells (*Neothauma tanganyicense*) on sandy bottoms, which they use for shelter and breeding, and occur in syntopic communities sharing large parts of their ecology (Lein and Jordan 2021). These fish maintain fixed home ranges centred on one or a few shells, which serve as their “home base” refuges (Gashagaza 1997; Lein and Jordan 2021). Some species within this group occur in areas of high shell density and may have home ranges of less than 1 square metre (Bose et al. 2020), while other species live in areas of open sand with few shelters available (Bills 1997). While species that live in high shell density areas may be able to find shelter easily, for those living in spare shell areas, knowing the exact location of each shell may be essential to avoiding predation. An individual’s shelter shell is a crucial resource and as such, we expect strong selection for homing in species that depend on one or a few unique shelters.

In the present study, we take a comparative natural history approach to spatial navigation in shell-dwelling cichlids. We first establish whether a standard displacement and return paradigm elicits comparable behavioural responses across different species, addressing a currently debated consideration in comparative cognition (Schuster 2025). Our field test leverages the fish’s natural motivation to return to its shelter, rather than using food rewards often employed in lab studies of fish cognition (Warburton 1990; Gómez-Laplaza and Gerlai 2011). This allows us to assess spatial memory in a natural context, providing ecologically relevant insight into their navigation strategies. We first census all shell-dwelling species in our study area to test whether they will navigate back to a particular shell after being experimentally displaced, or instead directly take shelter in the nearest available location. Then, for those species that do return to a particular location, we test for differences in their abilities to do so over increasing distances, and whether these differences are related to the size of their home ranges. Finally, in on shore arena experiments, we examine the role of landmarks in finding the location of a hidden shell. Rather than testing a single hypothesis, our goal is to provide a detailed comparative study that documents how navigation varies among species, and which ecological factors are most strongly associated with this variation.

## Methods

Fieldwork was conducted while SCUBA diving in the southern end of Lake Tanganyika (Zambia) between April 9 and May 30, 2022. Experiments were conducted at two sites on adjacent shores separated by ~ 10 km (Chikonde and Chitili). We selected seven species of shell-dwelling cichlid fishes (family Cichlidae, tribe Lamprologini): **(1)** *Telmatochromis temporalis*, **(2)** *Neolamprologus multifasciatus*, **(3)** *N. brevis*, **(4)** *N. pulcher* ‘Mwina’ (a population of dwarf *N. pulcher* that inhabits shells), **(5)** *Lamprologus ornatipinnis* **(6)** *L. ocellatus*, and **(7)** *N. meeli* (= *Lepidiolamprologus meeli* in some literature). All species occur in Chikonde, but because of differences in depth profiles and associated dive safety considerations, experiments were conducted at two field sites at equivalent depths of 8-12m. At both sites, substrate type, water depth, and temperature are equivalent. We did not take quantitative measurements of predator regimes, but visual inspection did not reveal obvious differences. At Chikonde (8°42’35.1” S, 31°07’22.4” E), we studied *L. ornatipinnis*, *N. brevis*, *N. multifasciatus*, *N. pulcher* ‘Mwina’, and *T. temporalis*, while at Chitili (8°38’53.9” S, 31°11’46.5” E) we studied *L. ocellatus* and *N. meeli*. All focal individuals were territory holders (the largest fish observed at the territory and aggressive to conspecific intruders). In this clade, this phenotype corresponds to dominant territorial males, a result verified in prior work in which individuals classified using these criteria were confirmed as males by gonadal inspection (Bose et al. 2025).

These species differ in how shells are distributed in their habitat and in typical home range size. *L. ornatipinnis*, *L. ocellatus*, and *N. meeli* inhabit areas with sparse shells surrounded by large stretches of open sand (Konings 2015; Sunobe and Munehara 2003). In contrast, *N. multifasciatus*, *N. brevis*, *N. pulcher* ‘Mwina’, and *Telmatochromis temporalis* inhabit shell bed habitats with high shell densities (Lein and Jordan 2021). To quantify these differences, we counted the number of visible shells within a 15 cm radius of the home shell (the shell into which the focal fish retreated), as well as the home range size (see below) for 10 individuals per species. These data are summarised in Table 1.

### Home range observations

Prior to performing displacements, we assessed the typical home range size of each species in situ. To estimate home ranges, we performed two different approaches, depending on the ecology of the species. For the species with small home ranges (*N. brevis*, *N. multifasciatus*,* N. pulcher* ‘Mwina’, and *T. temporalis*), we recorded the location of the dominant male and created a convex hull drawn around all observed points in which the fish swam during a 10-minute observation (following Bose et al. 2020). For the other species with larger home ranges (*L. ornatipinnis*, *L. ocellatus*, *N. meeli*) SCUBA divers followed focal individuals for approximately 5 min each, filming them from above with GoPro camera facing down to capture the fish’s movement area relative to fixed surroundings. We later reconstructed these videos into quantitative maps using an underwater structure-from-motion technique as described in detail in Francisco et al. (2020). Specifically, footage was processed with COLMAP (Schönberger and Frahm 2016), an open-source photogrammetry pipeline, to create a 3D point-cloud reconstruction of the scene. Depending on the conditions, RGB adjustments, contrast, brightness, black input levels, and sharpness on the videos were adjusted. From these reconstructions, we extracted the area over which the fish moved during observation, measured by outlining the convex hull of the camera positions in ImageJ (Rasband 1997). Home range estimates served two purposes: (1) to rank the species by relative range size (smallest to largest) for qualitative comparison to their navigation performance, and (2) to guide the displacement distances in our experiments and ensure displacements were beyond their home range boundaries. Existing literature values for home ranges were noted for context (Bills 1997; Sunobe and Munehara 2003), though these often refer to breeding territory (the area defended by the breeding pair against con- or heterospecific intrusion) and may not capture the full extent of home range.

### Field displacement experiments

**Fig. 1:**
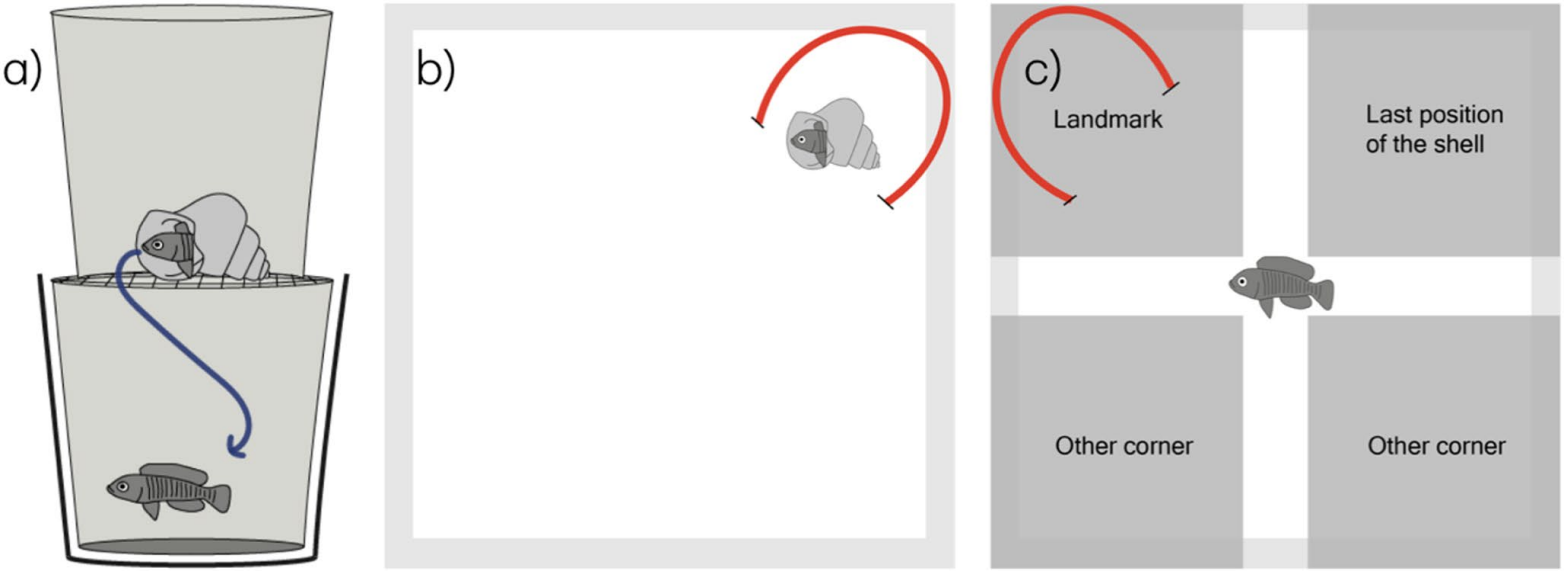
**(a**) Shell-extractor. Two transparent containers separated by a mesh grid large enough for the fish to pass through. When the individual is positioned in the upper chamber with the shell it swims down to the lower one, leaving the shell behind. When the individual is in the lower chamber, the shell is returned to its previous position in the sand and the dark container around the chamber is used for the displacement. **(b)** Training phase: a red string landmark is placed in association with the shell in which the focal individual shelters. **(c)** Testing phase. the dark grey corners represent the positions scored in the case that the individual enters that area

We first tested the response of each species to a minimal displacement of 30 cm from their home shell. A diver coaxed a focal fish into its home shell (by approaching slowly; the fish typically retreated inside its shell when disturbed) and then collected the shell containing the fish and placing it into a custom-made shell extraction device for 60–90 min (Fig. 1a). This device allowed us to separate the fish from its shell without harm. Once the fish was extracted inside the covered device, the empty shell was returned to its original location on the lake bottom. In these trials (*n* = 5 per species), we found that four species (*T. temporalis*, *N. multifasciatus*,* N. pulcher* ‘Mwina’, and *N. brevis*) did not return to their home shell after manipulation, instead choosing to hide in the shell bed where they were released until ushered back to their shelter by the experimenter. We therefore did not conduct further displacements for those species.

For the remaining three species (*L. ornatipinnis*, *L. ocellatus*, *N. meeli*), the fish was removed from its shell as above, the home shell returned to its original location, and a GoPro underwater camera was positioned at 50 cm of the home shell to record the return of the fish. Meanwhile, the diver transported the fish (enclosed in the dark container so it could not see the route or surroundings) to a pre-determined displacement distance from the home location. At the release point, the fish was released from the container and allowed to swim freely. The diver followed the fish from 2 to 3 m above with a camera to observe its movements after release (a focal follow video). If the fish swam out of view or could not be followed (e.g., due to turbidity or hiding among rocks or shells), the diver waited at the location of the last sighting for 20 min for the fish to reappear, and if this did not occur, abandoned the follow. The stationary camera at the home shell was left running for the remainder of the dive (~ 80 min total) to capture any returns that were otherwise not recorded during focal follows. As such, the return success for every fish was recorded for 80 min, and the subset of trials in which fish were successfully tracked could also be used to reconstruct the entire return path of the fish. Individuals were displaced 5 m, 10 m, or 15 m from their home shell. These distances range from being outside their typical home range (5 m) up to several times their usual range size (15–20 m) well into unfamiliar areas.

Each fish was tested only once (one displacement per individual) to maintain independent samples and avoid training effects. In total, we conducted 88 displacement trials for a target sample of *n* = 10 per species per distance. However, eventual sample sizes per species and distance varied due to the challenges of SCUBA diving based field biology (e.g., the diver lost sight of the fish in murky water, cameras flooded or overheated, cloud cover meant visibility became too low to resolve target fish in video) and those trials were excluded. We recorded two primary outcomes for each trial: (a) whether the fish successfully returned to its original shell, and (b) the time it took to return. A return was confirmed by the stationary camera footage showing the fish re-entering its home shell. If the fish did not return within the observation period, we recorded it as a navigation failure for that trial. The successful return of all tested individuals was confirmed by return observations within 48 h of each experiment. We also attempted displacements at 20 m but only three samples each were successfully collected for *L. ocellatus and L. ornatipinnis*, and one for *N. meeli*. Of these, only the single *N. meeli* was successful in returning to the home shell, and these data were not analysed any further.

From our trajectory reconstructions, we computed the path length and straight-line distance between the release point and the home shell. We defined a detour ratio as the path length divided by the straight-line distance (a measure of how indirect the route was). We also annotated these path videos for stops (pauses > 10 s) and social encounters (interactions with other fish along the way). Encounters were classified as meeting a heterospecific (different species), encountering a conspecific female, being chased or confronting a conspecific male, or encountering a large heterospecific (defined operationally as a fish at least twice the focal individual’s length, and therefore unlikely to be outcompeted in a dispute). These observations provide qualitative context of the external factors that might influence navigation success or speed (e.g., stopping to hide from a large heterospecific or being delayed by border conflicts).

For statistical analysis of the field displacement experiment, we restricted inference to the three species that reliably expressed homing under the assay in pilot trials (*Lamprologus ornatipinnis*,* L. ocellatus*,* Neolamprologus meeli*), and to the displacement distances for which these species were tested with replicated trials (5, 10, 15 m). Each trial was scored as a binary outcome indicating whether the focal individual re-entered its original home shell within the observation window (success vs. failure), as confirmed by the stationary camera at the home shell. We analysed homing success across distances using a trial-level binomial logistic regression with fixed effects of displacement distance (treated as a continuous predictor in metres), species (categorical), and their interaction (Success ~ Distance × Species). This model was used to test whether the probability of successful homing declined with distance, and whether species differed in baseline success or in the distance-dependence of success, using standard model-based tests for the distance main effect, the species main effect, and the species × distance interaction. Because only three species contributed distance-series data, and because the analysis is conducted within a small clade with limited degrees of freedom, no phylogenetic correction was applied.

Because the raw data indicated that species divergence was most pronounced at the maximal displacement distance (15 m), we additionally tested for an overall difference among species in success at 15 m using an omnibus Fisher–Freeman–Halton exact test on the 3 × 2 contingency table (species × success/failure), implemented by enumeration of all tables with fixed margins. We then performed pairwise, two-sided Fisher exact tests between species at 15 m. We did not include home range size or local shell availability as predictors in the homing-success models, because these ecological variables are species-level quantities that are monotonic with the observed species ordering in homing performance among the three navigating species.

Next, we analysed return time for those individuals that successfully returned home. Return time (log-transformed to meet normality assumptions) was compared across species and distances using a two-way ANOVA (species, distance). We also performed a one-way ANOVA for the effect of displacement distance (5 vs. 10 vs. 15 m) pooled across species to see if further displacements generally took longer to home. ANOVA assumptions (normality of residuals via Shapiro-Wilk test, homogeneity of variances via Levene’s test) were satisfied.

For the path analysis of successful trials (*n* = 15 total across species), we compared the detour ratio among species using a non-parametric Kruskal-Wallis test and the frequency of different encounter types among species using chi-square tests. Finally, we used multiple regression whether any of the observed factors (encounters, distance, species) significantly predicted return time for those fish that did return. An ordinary least squares (OLS) linear regression was constructed with return time as response and predictors: distance displaced, number of large heterospecific encounters, number of conspecific male encounters, etc., and species (as categorical). Diagnostics (Breusch-Pagan test for heteroscedasticity, Durbin-Watson for independence, residual plots for linearity, Shapiro-Wilk for normality of errors) were performed to ensure validity of the linear model. We did not include home range size or shell abundance as predictors because this would yield coefficients that perfectly restate the rank ordering among the three species and would therefore provide no additional inferential power. With only three species contributing quantitative homing data, such models are mathematically underdetermined and risk overinterpretation. Our experimental design prioritized ecological validity by testing wild fish in their natural environment using their intrinsic motivation to return to a shelter. This approach allowed us to assess navigation under naturalistic conditions but cannot manipulate potential factors shaping cognition. We therefore treat our results as documenting associations between ecological context and navigation behaviour rather than demonstrating causation.

### Shore experiments

To further probe the navigation mechanisms, we conducted laboratory experiments with the species that showed effective homing in the field (*L. ornatipinnis*, *L. ocellatus*, *N. meeli*). These tests were performed in arenas immediately adjacent to the lake shore at Kalambo Falls Lodge (on Lake Tanganyika) using wild-caught dominant males of the three species (*N* = 11 *L. ornatipinnis*, 12 *L. ocellatus*, 12 *N. meeli*). Fish were kept in holding tanks of 30–40 individuals for 7–8 days to acclimate in mixed sex groups and then tested individually. After experiments, all fish were released back to their capture sites.

The arena setup was a square concrete tank (approximately 90 cm × 90 cm floor, 60–80 cm water depth) with uniform sand substrate and white walls to minimize the effect of uncontrolled landmarks. In one corner of the tank, we placed a 3D-printed replica snail shell (similar size to a real *Neothauma* shell) which served as the “home shell” surrogate. A distinctive visual cue (a red nylon string) was introduced as a landmark. The string was stretched across the sand from one wall to the adjacent wall, passing under the shell, such that the shell sat at the midpoint of a red line (the middle section of string was slightly elevated, forming an arch over the sand) and was thus an obvious visual cue in an otherwise featureless tank (Fig. 1b).

Each fish was given 24 h to accept the artificial shell as a shelter, and we only proceeded with experiments if fish consistently hid in the shell when disturbed to ensure the shell had salience as the home shelter. We then carried out four training trials followed by a test trial for each fish. In each training phase, the fish was captured in a small container and placed into the centre of the tank (while inside a covered shell-extractor device to reduce stress). The sand substrate was smoothed flat before each trial to remove any visual cues on the sand from previous trials. We relocated the shell and landmark at the start of each training phase: the shell (with its red string) was moved to a different corner of the tank for each phase, in random order, so that the fish experienced the shell in multiple corners (always associated with the red string marking its position). This process ensured that the experimental landmark was dissociated from any incidental cues on the tank walls and ensured that the red string was the only landmark consistently associated with the shell. When the fish was released in the centre, it was allowed to explore and search for the shell. The position of shells in the training phase was switched before departing for diving (between 07:00–08:00) or after returning from diving (16:00–17:00) and then left undisturbed until the next phase. Half the trials began in the morning and therefore proceeded AM: PM: AM: PM and half PM: AM: PM: AM, such that every focal individual experienced two AM and two PM training phases. After four training phases we conducted a test phase at either 08:00 or 18:00, removing the shell from the tank entirely, and leaving only the red string landmark (placed in a new corner). The fish was again released in the centre under cover and then allowed to explore the tank for 20 min. We recorded the fish’s behaviour on video during the test. We measured whether the fish would search for the shell in its last known location, in association with the landmark, or in random areas of the tank.

For analysis of the test videos, we divided the tank into four response areas with a no-choice buffer area (Fig. 1, right panel). We scored which area the fish entered and recorded: (i) the first area it swam to after release (once it started actively exploring), and (ii) the area where it spent the longest duration over the 20 min test. If a fish was in the central area (not in a corner zone), that time was not attributed to any corner. For each species, we compared the number of first choices directed toward shell-associated areas (last known location of the shell + landmark combined) against those directed toward other areas using exact binomial tests (null = 0.5). For species with a significant bias, we then conducted a second binomial test comparing landmark versus last known location of the shell first choices. Because this second test is logically dependent on the first, no additional multiplicity correction was applied. To test whether fish preferentially occupied shell-associated areas, we used a prespecified, two-stage procedure; first, for each species, we calculated the difference in time spent in shell-associated areas (last known location of the shell + landmark) versus other areas per replicate and tested whether the median difference exceeded zero using a Wilcoxon signed-rank test. To account for multiple species, we applied Holm’s sequential correction across the three species’ Stage-1 tests. Subsequently, for species with a significant Stage-1 result, we tested whether time spent at landmarks differed from time spent at the last known locations of the shell using a Wilcoxon signed-rank test. Because this test was contingent on a significant Stage-1 effect and represents a planned decomposition of the primary effect, no further multiplicity correction was applied. Effect sizes for paired comparisons were expressed as Cohen’s dz (mean difference divided by the SD of differences). All analyses were performed in Python 3.11.

## Results

### Home range size and shell density

**Table 1:** The mean and median number of visible shells within a 15 cm radius of the focal individual’s home shell and home range area for each of the seven Lamprologine cichlids in this study (*n* = 10 per species)

Species	Shells (mean ± SD)	Shells (median)	Home range cm² (mean ± SD)	Home range cm² (median)
*T. temporalis*	9.4 ± 2.4	9.0	72.5 ± 10.8	72.0
*N. multifasciatus*	9.0 ± 2.2	9.0	79.6 ± 15.6	78.5
*N. pulcher*	11.4 ± 1.8	11.0	150.9 ± 74.5	154.0
*N. brevis*	12.6 ± 2.3	12.0	189.8 ± 74.6	183.0
*L. ornatipinnis*	1.0 ± 0.0	1.0	609.7 ± 168.5	617.0
*L. ocellatus*	1.0 ± 0.0	1.0	1 016.6 ± 462.9	1 013.0
*N. meeli*	2.4 ± 1.0	2.0	1 803.5 ± 867.7	1 879.0

We found distinct differences among our study species in home range size, broadly divided into two groups; *N. multifasciatus*,* N. brevis*,* N. pulcher* ‘Mwina’, and *T. temporalis* had the smallest ranges with all individuals occupying home ranges of less than 200 cm^2^, a result consistent with findings for *N. multifasciatus* using both automated tracking (Bose et al. 2020) and with genetic analyses (Bose et al. 2022) and consistent with qualitative descriptions for other species (Bills 1997; Konings 2015). These species with relatively smaller home ranges had 8–12 visible shells in their territories. By contrast, the other three species had larger home ranges and 1–2 shells in the immediate vicinity; *L. ornatipinnis* had a mean home range area of 609.7 cm², *L. ocellatus* had a larger median range area 1016.6 cm², and *N. meeli* exhibited the largest range: median area 1803.5 cm² (Table 1). Our estimates, measured directly by tracking fish, are likely more accurate than previous reports in the hobbyist literature (Konings 2015) as they capture both the defended space as well as exploratory behaviour.

### Displacement experiment

After having been displaced from their home shells, the four species with the smallest ranges, *N. multifasciatus*,* N. brevis*,* N. pulcher* ‘Mwina’ and *T. temporalis* did not return home even from small displacement distances (30 cm). Instead, they would quickly shelter under the nearest shell or rock and remain there until they were shepherded back to their original shell by the experimenter. Given their small size and high predation risk without a shell, it appears these species, among the smallest cichlids in Lake Tanganyika, prioritized immediate shelter over homing, especially when isolated. We therefore withhold judgement about their navigational capacities, concluding that alternative designs are needed to adequately assess their spatial cognition.

**Fig. 2:**
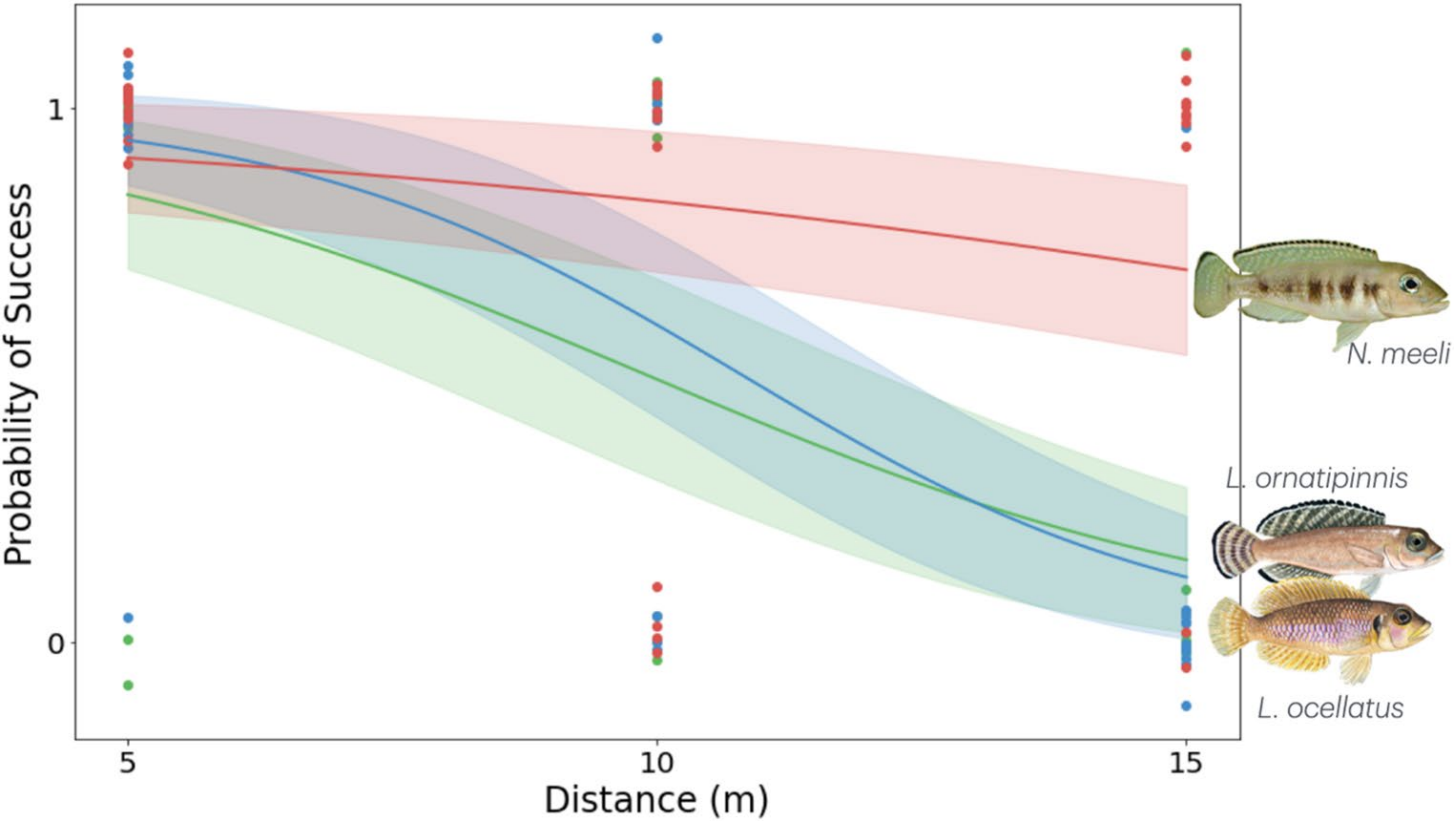
Logistic regression and confidence intervals by species. Probability of returning to home shell (1 = returned, 0 = did not return) at three displacement distances (5, 10, 15 m from home shell) for three species: *Neolamprologus meeli* (red), *Lamprologus ocellatus* (blue), *Lamprologus ornatipinnis* (green). A significant reduction in the likelihood of successful return with increasing distance was observed for *L. ocellatus* and *L. ornatipinnis*. At 15 m, *N. meeli* returned significantly more frequently than *L. ornatipinnis* or *L. ocellatus*

In the three species *L. ornatipinnis*, *L. ocellatus*, and *N. meeli*, navigation performance was related to the distance displaced (binomial logistic regression, distance main effect: χ²(1) = 9.60, *p* = 0.00194; Fig. 2). Across all distances, there was no evidence for a uniform species effect on success (species main effect: χ²(2) = 1.00, *p* = 0.606), and the species × distance interaction was not significant (χ²(2) = 3.03, *p* = 0.220), indicating limited power to resolve differences in the shape of success–distance curves across the full 5–15 m range.

We then compared performance at the maximal displacement distance of 15 m, at which *N. meeli* returned in 8/10 trials (0.80), while *L. ornatipinnis* returned in 1/10 trials (0.10) and *L. ocellatus* returned in 1/11 trials (0.09). An omnibus Fisher–Freeman–Halton exact test confirmed that success differed among species at 15 m (*p* = 0.000461). Pairwise Fisher exact tests indicated that *N. meeli* was significantly more successful than both *L. ocellatus* (*p* = 0.00191; odds ratio = 40.0) and *L. ornatipinnis* (*p* = 0.00548; odds ratio = 36.0), whereas *L. ocellatus* and *L. ornatipinnis* did not differ (*p* = 1.00; odds ratio = 0.90). Together, these analyses show a robust overall decline in homing success with distance, alongside species differences in long-distance homing robustness at 15 m, driven by the sustained performance of *N. meeli*.

These patterns demonstrate clear species-level differences in navigation behaviour under our assay conditions. It is important to note that among the three navigating species variation in long-distance homing success (particularly at 15 m) was perfectly rank-ordered with both home range size and local shell scarcity, such that the species with the largest range and sparsest shelters (*N. meeli*) showed the highest success, while the two species with smaller ranges and somewhat greater shell availability (*L. ocellatus*,* L. ornatipinnis*) showed lower success. This perfect covariation among ecological variables prevents formal statistical partitioning of their independent effects, but provides a strong descriptive pattern for future investigation.

**Fig. 3:**
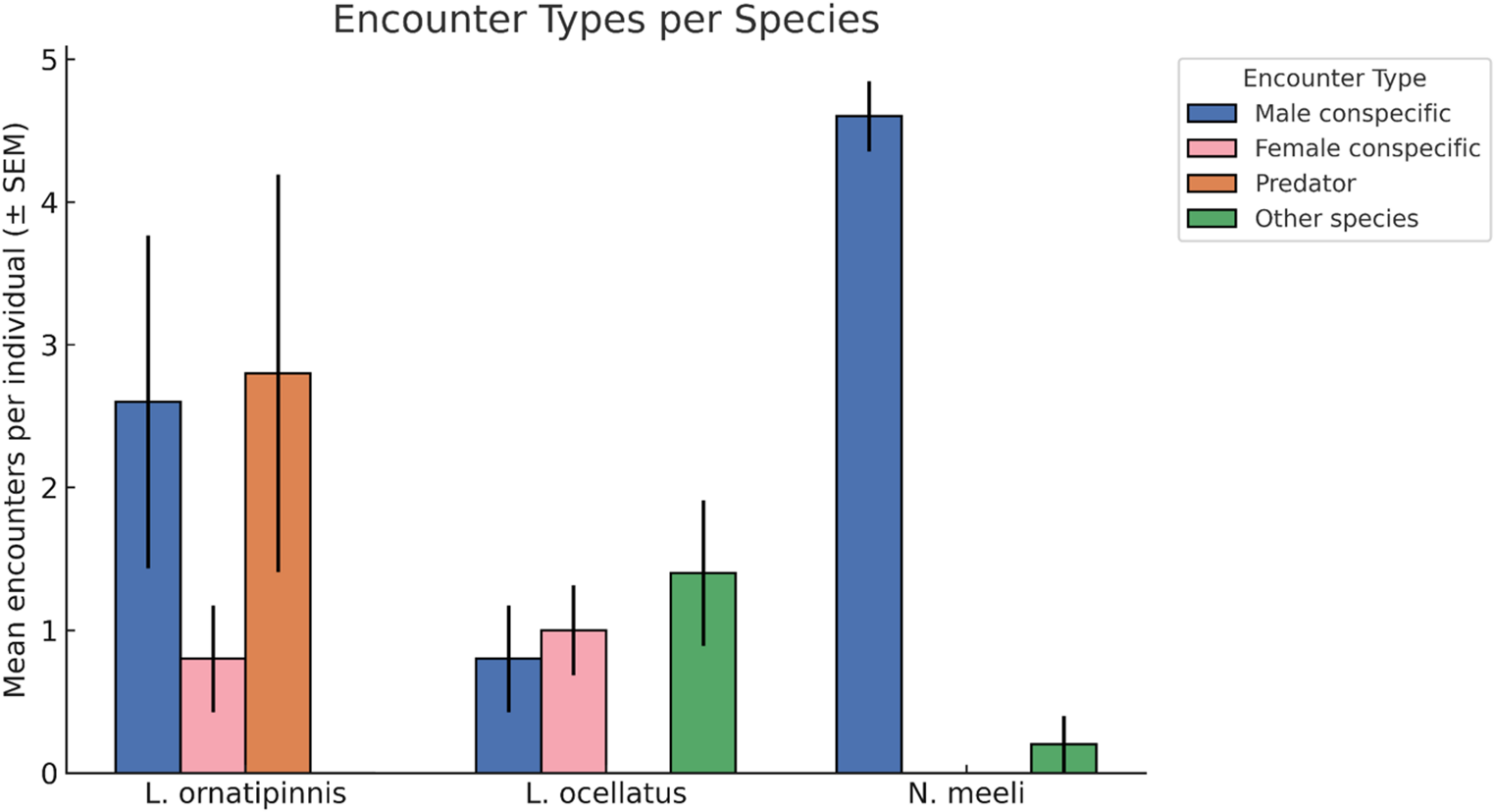
Encounter types per species. Mean number of encounters per individual (± SEM) across species. Each cluster of bars corresponds to one species (*L. ornatipinnis*,* L. ocellatus*,* N. meeli)*. Within species, bars represent different encounter types: male conspecific (dark blue), female conspecific (rose), large heterospecific (orange), and other species (green)

The distribution of return times (for those individuals that did return, *N* = 54 trials) was highly skewed (many fast returns and a few very slow); data were therefore log-transformed for analysis. There was no significant difference in return time between species, and no species × distance interaction (two-way ANOVA, *F* < 1, *p* = 0.77 for interaction; main effect of species: *p* = 0.40). The main predictor of return time was distance: pooling species, fish displaced further took longer to return (one-way ANOVA on distance: *F*2,51 = 8.73, *p* = 0.0005). Post-hoc Tukey tests confirmed a significant difference between 5 m vs. 15 m (pairwise *p* = 0.001), whereas 10 m was intermediate and not significantly different from 5 to 15 at α = 0.05. Specifically, median return times were ~ 2 min for 5 m, ~ 5–6 min for 10 m, and ~ 10 + minutes for 15 m displacements (with substantial variation) Fig. 3.

We next inspected the path trajectories of individuals that returned to elucidate potential navigation strategies. Reconstructed path maps for each species (Supplementary Figures S1, S2, S3) revealed that fish took circuitous routes. The ratio of actual path distance to the straight-line distance ranged from near 1.0 (almost straight) up to ~ 2.5 (significant detours), with an average around 1.5–1.8. There was no significant difference in this detour ratio between *L. ornatipinnis*, *L. ocellatus*, and *N. meeli* (Kruskal-Wallis *H* = 0.5, *p* = 0.78).

During these return journeys, we observed a variety of social interactions, the frequencies of which differed by species (χ² test of encounter type counts vs. species, *p* < 0.001). *L. ornatipinnis* individuals, for example, had a higher incidence of large heterospecific encounters along the way (in 4 of 5 mapped paths). *N. meeli* experienced frequent chases and aggression from conspecific males when crossing into others’ territories: in most *N. meeli* returns, the homing individual was intercepted and chased multiple times by resident males whose territories it trespassed. *L. ocellatus*, on the other hand, had relatively few encounters of any kind. Notably, *L. ocellatus* also paused (stopped moving for > 10 s) more often than the others (27 total stops observed across its 5 paths, vs. 7 in *L. ornatipinnis* and 4 in *N. meeli*), some of which were stops near conspecific females’ shells.

Finally, we explored if these encounters and detours had measurable effects on return time. The OLS regression on return time (for the 15 successful path-mapped trials) including predictors (distance, large heterospecific encounters, conspecific chases, etc.) explained 59% of variance (R² = 0.59) but was below significance threshold (*p* = 0.0998). One factor was significant: the number of large heterospecific encounters increased return time (β ≈ +58 s per large heterospecific encounter, *p* = 0.043), and there was also a trend for male-male conspecific encounters (chases by territorial males) to correlate with shorter return time (β ≈ − 62 s per chase, *p* = 0.064).

### Shore experiment results

Both *L. ocellatus* (*p* = 0.019) and *N. meeli* (*p* = 0.00024) were significantly more likely to make their first choice toward a shell-associated area, whereas *L. ornatipinnis* did not differ from chance (*p* = 0.50). Among individuals that chose a shell-associated area first, planned comparisons showed no species showed a significant preference for landmarks over last known locations of the shell (all binomial *p* > 0.34).

**Fig. 4:**
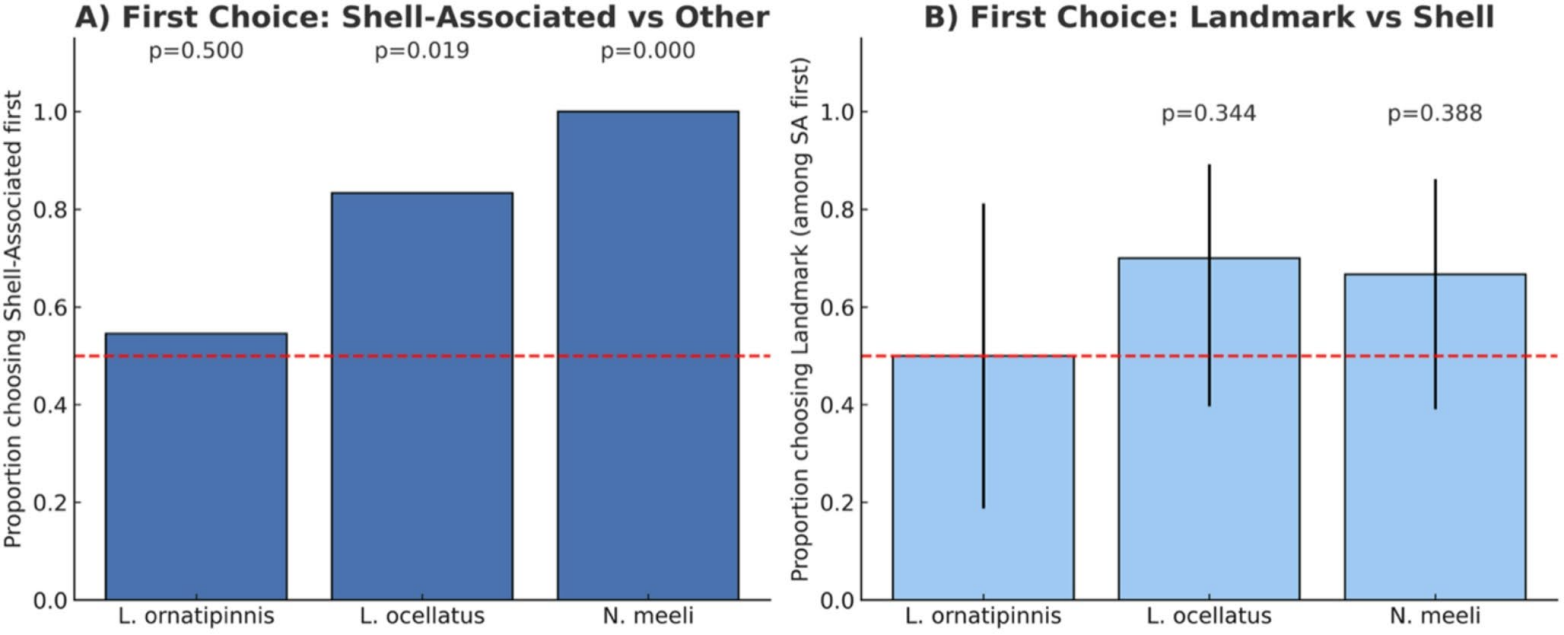
First-choice behaviour. (**A**) Proportion of first choices directed toward shell-associated areas (shell + landmark combined) for each species. The dashed red line indicates chance expectation (0.5). Exact binomial p-values are shown above bars, aligned on a uniform horizontal line for comparability across species. (**B**) Proportion of shell-associated first choices that were directed toward landmarks (vs. shells – meaning last known location of the shell), with Wilson 95% confidence intervals. The dashed red line indicates no preference (0.5). Exact binomial p-values are displayed above error bars, aligned on a uniform line

**Fig. 5:**
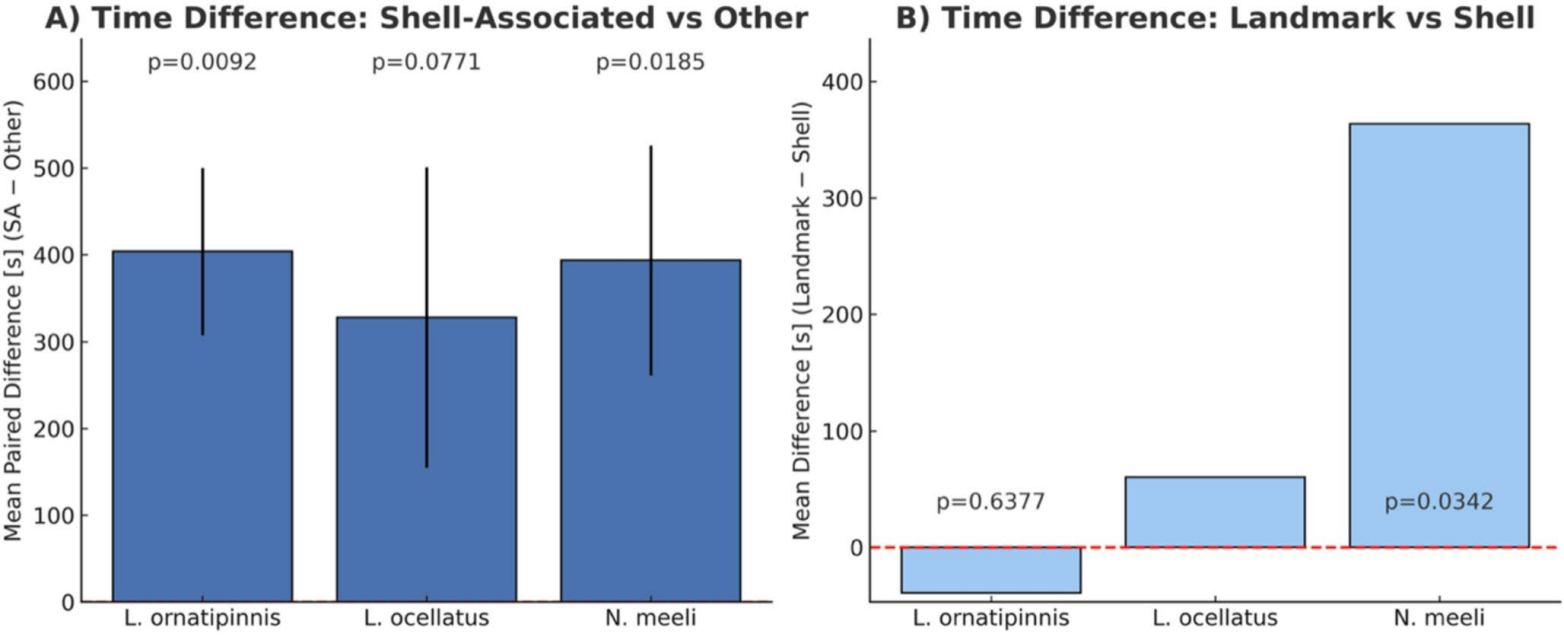
Time allocation differences. (**A**) Mean paired difference (± SEM) in time spent in shell-associated areas (last known location of the shell + landmark) compared to other areas (SA − Other). Positive values indicate more time in shell-associated areas. The dashed red line represents no difference. Wilcoxon signed-rank p-values are shown above bars, aligned on a uniform line for all species. (**B**) Mean paired difference (± SEM) in time spent at landmarks compared to shells (Landmark − Shell) – Shell meaning last known location of the shell. Positive values indicate greater landmark use. Wilcoxon signed-rank p-values are shown above the x-axis baseline on a uniform line across species

Both *L. ornatipinnis* (*p* = 0.009) and *N. meeli* (*p* = 0.018) spent significantly more time in shell-associated areas compared to other areas; these results remained significant after Holm’s correction across species (Wilcoxon test; adjusted *p* = 0.027 and 0.036, respectively). *L. ocellatus* showed a non-significant trend (*p* = 0.077). Of the species that spent significantly more time in an area previously associated with a shell, planned comparisons showed that *N. meeli* spent significantly more time at landmarks than at the last known locations of the shell (Wilcoxon test; *p* = 0.034), whereas *L. ornatipinnis* showed no significant preference (*p* = 0.638) Figs. 4 and 5.

## Discussion

We explored variation in spatial cognitive abilities in Lake Tanganyikan cichlids under natural conditions, finding that a standard paradigm was not equally applicable to even closely related species that share much of their ecology; of the 7 species tested, only 3 species reliably returned to their home shelter after displacement. This suggests that even in relatively straightforward experimental designs – here simply moving an animal to a new location – small differences in ecology among species can influence the efficacy of the paradigm. Despite being highly philopatric and maintaining fixed territories in space (Jordan et al. 2020; Bose et al. 2020), four of the species tested sought immediate shelter rather than returning to their home shelters. In contrast, for the 3 species that did reliably return to their home shells, we found the ability to navigate back to their shells decreased with distance, and that species differed in their ability to navigate back once displacement distances extended to 15 m. Moreover, the species with the largest home range (*Neolamprologus meeli*) showed substantially greater long-distance homing success, a result consistent with predictions from the range size hypothesis. Onshore experiments mirrored these patterns, with the species that were better at finding their home shells also demonstrating targeted searching for their missing shells in the arena. These findings provide the first detailed comparative analysis of spatial navigation across multiple fish species tested under standardized natural conditions, showing variation even among phylogenetically close species that share many aspects of their ecology.

The ecological contrast between species occupying sparse-shell habitats and those living in dense shell beds offers insight into the selective pressures that may shape interspecific behavioural differences. For species such as *N. meeli*,* Lamprologus ocellatus*, and *L. ornatipinnis*, each home shell represents a rare resource in an otherwise open sandy environment. Failure to return to one’s particular shell after foraging or being displaced could mean permanent loss of breeding territory and heightened vulnerability to predators. In such conditions, selection is expected to favour individuals motivated to return to their home shells and capable of remembering precise locations and navigating to them accurately. Consistent with this expectation, the species with the largest home range also showed the greatest navigation success after displacement. While our design does not allow a formal test of the range size hypothesis (because home range size, habitat structure, and navigation performance covary perfectly among the navigating species, rendering their independent effects statistically non-identifiable) the observed pattern remains consistent with the prediction that spatial cognitive performance scales with the spatial demands imposed by a species’ natural movement ecology. The role of social and ecological interactions during the displacement trials also highlights the real-world consequences of navigation under natural conditions. During their path, some individuals interacted with and were potentially delayed or diverted by large heterospecific or conspecific rivals. Large heterospecific encounters, i.e. fish that could only be avoided but not challenged, were associated with greater return times, while aggressive chases sometimes appeared to redirect displaced individuals back towards their home shell. These interactions illustrate that navigation in the field is embedded within a web of ecological pressures, and performance may be due to both cognitive capacity as well as decision-making in a risky environment.

For species such as *Telmatochromis temporalis*,* N. multifasciatus*,* N. pulcher* “Mwina”, and *N. brevis*, shells occur in high densities, and individuals are rarely far from alternative refuges. In this context, rapid use of the nearest available shell may be a safer strategy than attempting to return to a specific one, especially under the immediate threat of predation. Our natural experiment, aimed at studying cognition in the wild, cannot isolate navigational capacity from motivational differences and risk assessment. Importantly, these factors are not mutually exclusive and under natural conditions likely interact in this and most other systems. A species may for instance possess the cognitive capacity for navigation but lack the ecological incentive to express it (as we likely observed in shell-bed species). The behaviour of all species in our study therefore likely reflects a different balance between motivational state and risk management, rather than being evidence of an absence of spatial information per se. Future studies must therefore carefully consider how cognitive assays, especially in the wild, interact with both a species cognitive capacities and their motivation to express these in a given experimental paradigm (Schuster 2025; Greggor et al. 2015). We hope that our study also underscores the value of natural history observations and pilot testing, ideally under natural conditions, to determine whether a given assay elicits comparable behavioural responses across species before interpreting differences as cognitive variation. Indeed, the challenge of separating motivation from ability is not unique to Tanganyikan cichlids, or to fish. Performance differences can reflect differences in cognitive capacity, but also differences in sensory abilities, motor skills, neophobia, stress responses, or the salience of experimental stimuli (Morand-Ferron et al. 2016). Although we harnessed the motivation of these shell-dwelling species to return to their shells as one solution to the problem of motivation, our results showed that even this seemingly fundamental behaviour may vary across species due to ecological context.

In addition to demonstrating variation in navigation abilities, our results also offer insight into the mechanisms of navigation employed by the homing species. Return trajectories were rarely straight, but instead included detours, pauses, and apparent searching. This pattern suggests that fish were not relying on path integration (i.e. updating direction relative to a starting point by integrating information about the distance and direction travelled; Etienne and Jeffery 2004) but instead on memory of local landmarks or spatial configurations. In our arena experiments, both *N. meeli* and *L. ornatipinnis* spent significantly more time in shell-associated areas than in other areas, indicating that these species actively seek out features of the shell environment when navigating. Moreover, *N. meeli* showed a specific preference for the landmark over the last known location of the shell, suggesting that it may prioritise prominent spatial cues during navigation. *L. ornatipinnis*, by contrast, did not distinguish between the landmark and the last known location of the shell, indicating a broader reliance on shell-associated space. Interestingly, *L. ocellatus* was more likely than expected to make its first choice toward a shell-associated area, but it did not subsequently spend more time there overall. This discrepancy between initial choices and sustained allocation of time highlights that different components of navigation behaviour may be underpinned by distinct mechanisms. These patterns mirror findings in other fishes and invertebrates, where landmark-based navigation can complement or substitute for compass orientation (Wehner 2003; Cheng 2014). In the wild, species inhabiting open habitats may rely on sparse but reliable visual features such as rocks or vegetation to navigate (Wehner 2003).

Because we cannot statistically partition the effects of range size, shelter availability, predation pressure, and motivational state, as these covary among species, experimental manipulations will be required to elucidate the role of each in shaping spatial cognition. While temporary removals of e.g. all but the home shell from high-density areas, or adding shelters to sparse areas could help isolate mechanisms, previous manipulations of shell density have shown large, and unexpected changes in both social organisation and predation risk (Jordan et al. 2016). In addition, we focused exclusively on territorial males that were easily identifiable, but females and subordinates may show different navigation capacities depending on their ranging behaviour, resource needs, and philopatry (e.g. Perdue et al. 2011). Predation pressure may also play a role in shaping the risk environment, and therefore the costs and benefits of spatial cognition, and is known to affect group composition in Lamprologine cichlids (Groenewoud et al. 2016), and could also be usefully studied in future work. Despite these limitations, our study provides perhaps the most comprehensive comparative dataset on fish spatial navigation under natural conditions to date, demonstrating that substantial cognitive variation exists among species that are phylogenetically close and ecologically similar. Future work that directly manipulates the factors we have identified here, directly compares sexes and life stages within species, and expands to broader taxonomic sampling, will help determine which aspects of spatial ecology most strongly shape cognitive evolution.

Our study of navigation in wild cichlids showed their performance was strongly associated with home range and shelter availability: species with larger ranges and sparse shelters reliably homed across distances exceeding 15 m, while species with smaller ranges and abundant shelters prioritized immediate refuge-seeking over returning to a specific shelter. These patterns are consistent with spatial cognition being shaped by species-specific ecological demands, though further work is needed to distinguish the exact contribution of navigational capacity, motivational state, and adaptive risk in shaping spatial cognition. Nevertheless, our findings contribute to a growing body of evidence that cognitive traits vary predictably with ecology (Spencer 2012; Healy and Rowe 2007), and highlight the dual challenges of isolating causal mechanisms in natural systems and applying standardised tests in the wild. By providing detailed comparative data on navigation across multiple species under ecologically valid conditions, this work establishes a foundation for future experimental investigations into the mechanisms that link ecology and spatial cognition in one of the world’s most diverse radiations.

## Supplementary Information

Below is the link to the electronic supplementary material.

Supplementary Material 1

## Data Availability

Data and code are available as supplementary files.
