# Supplementary material for: Comparative spatial cognition in wild Tanganyikan cichlids: navigation performance varies with home range and shelter availability
Source: Anim Cogn. 2026 Apr 13;29(1):38. doi: 10.1007/s10071-026-02064-2 (PMC13083442; doi:10.1007/s10071-026-02064-2)
Supplement: Supplementary file 1 — Supplementary Material 1 [file 10071_2026_2064_MOESM1_ESM.docx]

**Supplementary Figures**


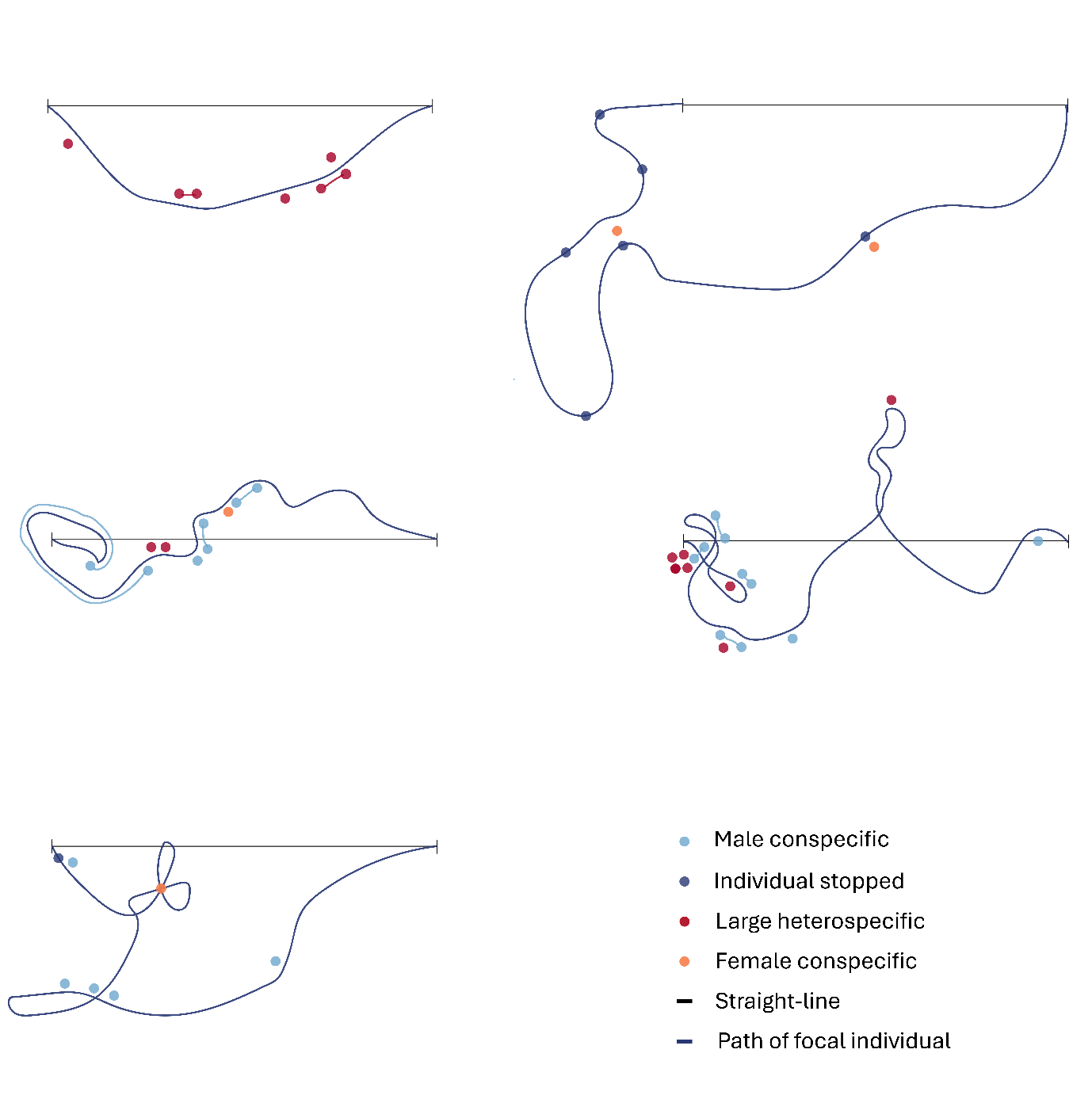


Figure S1: Visual representations of paths of *Lamprologus ornatipinnis* returning to their shell. The dark blue line is the path of the focal fish, and the black line illustrates the shortest path to home shell. Circles represent encounters with other individuals and additional matching lines represent a follow by the other individual. Each path begins on the left with the end point being the home shell on the right. Paths 1, 2 and 3 are individuals that were displaced 5 metres. 4 and 5 displaced 10 metres.


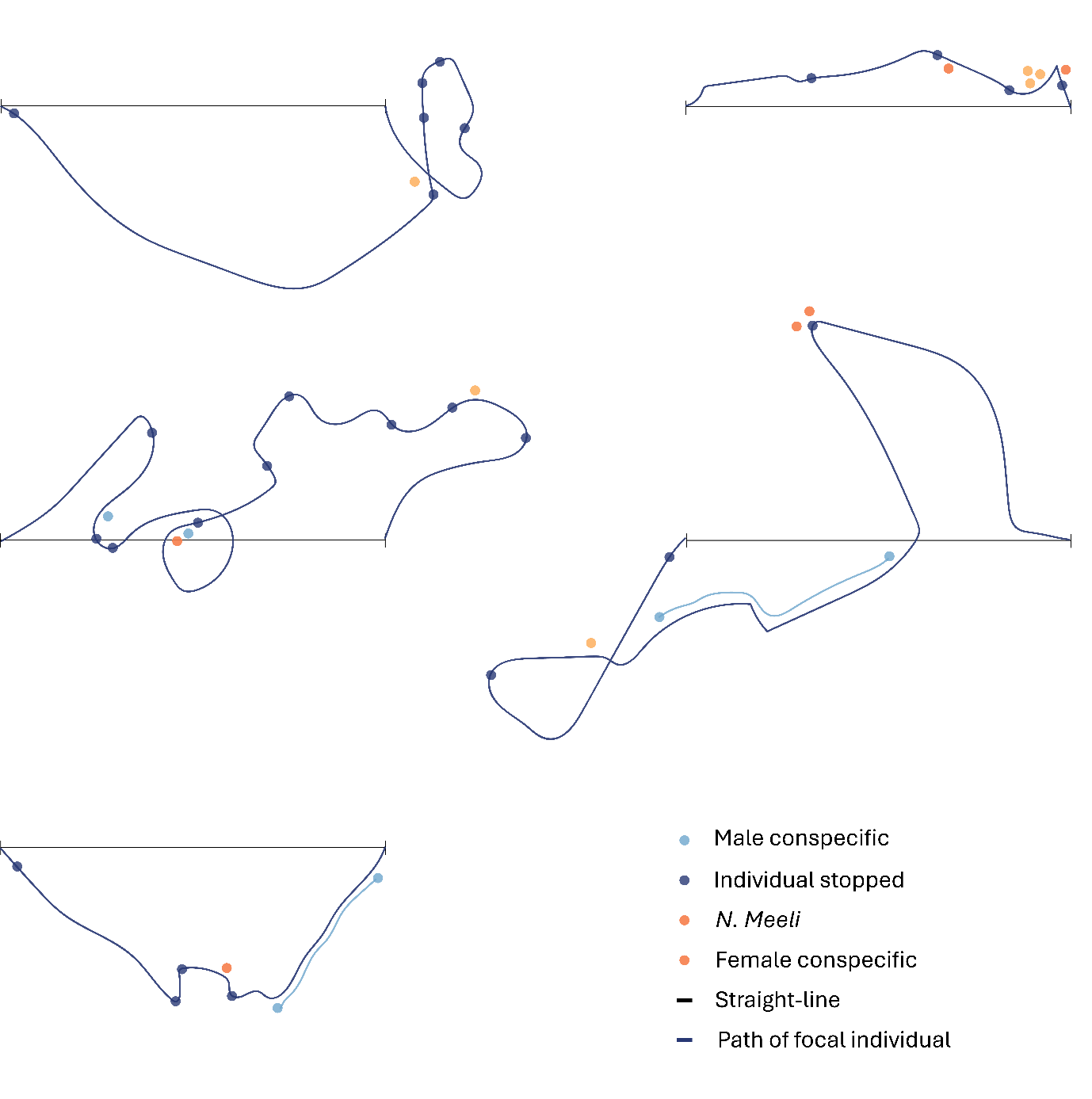


Figure S2: Visual representations of paths of *Lamprologus ocellatus* returning to their shell. The dark blue line is the path of the focal fish, and the black line illustrates the shortest path to said shell. Circles represent encounters with other individuals and additional matching lines represent a follow by the other individual. Each path begins on the left with the end point being the home shell on the right. Paths 1, 2 and 3 are individuals that were displaced 5 metres. 4 and 5 displaced 10 metres.


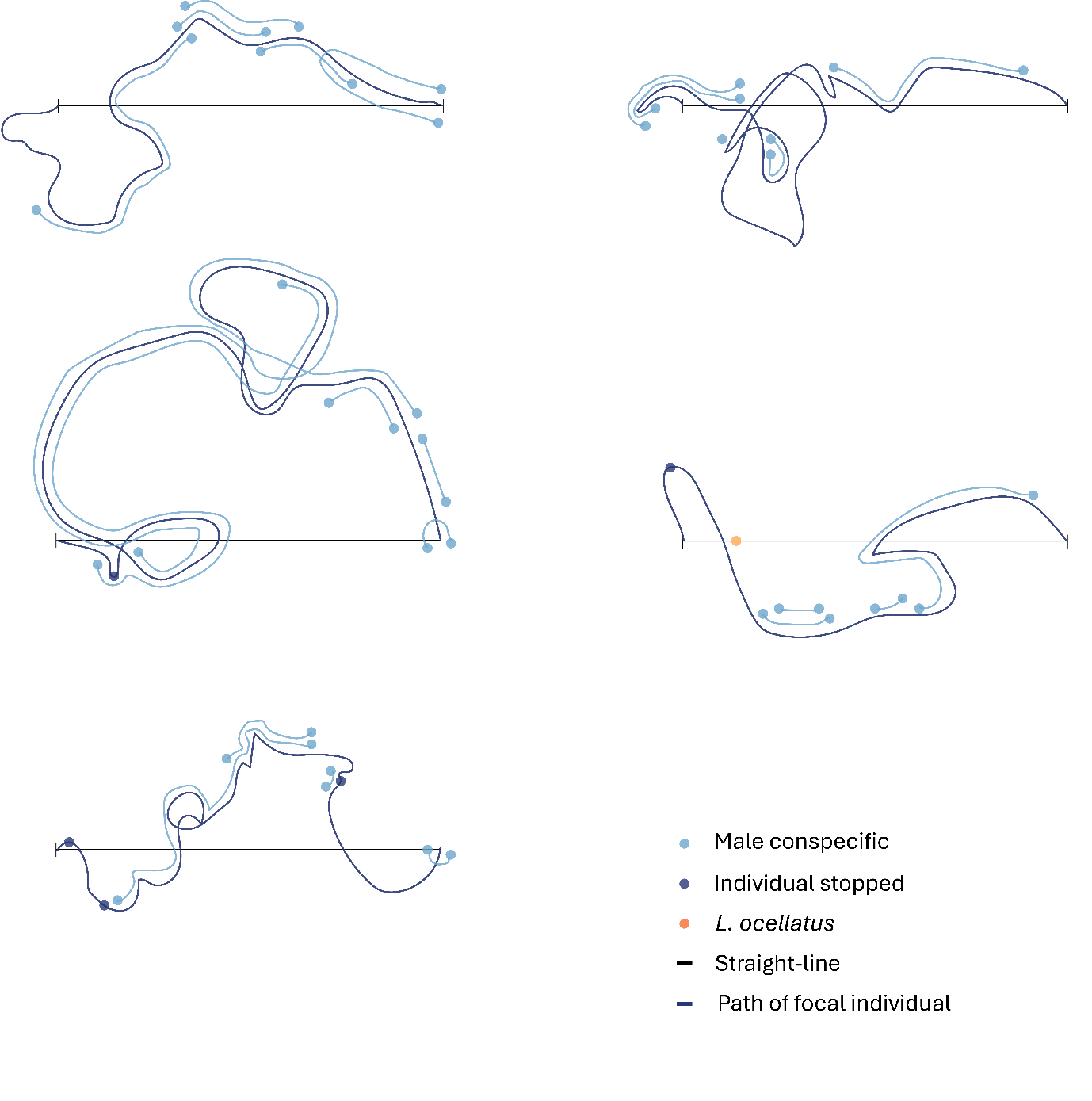


Figure S3: Visual representations of paths of *Neolamprologus meeli* returning to their shell. The dark blue line is the path of the focal fish, and the black line illustrates the shortest path to said shell. Circles represent encounters with other individuals and additional matching lines represent a follow by the other individual. Each path begins on the left with the end point being the home shell on the right. Paths 1, 2 and 3 are individuals that were displaced 5 metres. 4 and 5 displaced 10 metres.
